# Protocol for isolating leukemia-derived extracellular vesicles from the spleen of preclinical models of leukemia using ultracentrifugation

**DOI:** 10.1016/j.xpro.2024.103244

**Published:** 2024-08-05

**Authors:** Ernesto Gargiulo, Pablo Elias Morande, Maxmilan Jeyakumar, Lucie Rospape, Jérôme Paggetti, Etienne Moussay

**Affiliations:** 1 Tumor Stroma Interactions, Department of Cancer Research, Luxembourg Institute of Health, L-1210 Luxembourg, Luxembourg; 2 Instituto de Medicina Experimental (IMEX)-CONICET, Academia Nacional de Medicina, Buenos Aires, Argentina; 3 Faculty of Science, Technology and Medicine, University of Luxembourg, Esch-sur-Alzette, Luxembourg

**Keywords:** cell biology, cell separation/fractionation, cancer

## Abstract

Here, we present a protocol for the direct isolation of small extracellular vesicles (sEVs) from the spleen of preclinical murine models of leukemia using ultracentrifugation. We describe steps for tissue collection, sample preparation, ultracentrifugation-based isolation, and sEV characterization. This protocol allows for efficient enrichment of both leukemia and its microenvironment-derived sEV (LME-sEV), providing a valuable tool for studying their composition and functional roles. Potential applications include investigating the role of sEV in leukemia progression and identifying biomarkers.

For complete details on the use and execution of this protocol, please refer to Gargiulo et al.^1^

## Highlights

• Isolation of sEV from the spleen of preclinical murine models of leukemia
• Full representation of sEV produced in the tumor microenvironment
• Characterization by immunoblotting, microscopy, size determination, and flow cytometry

## Abstract (editor highlights)

Publisher’s note: Undertaking any experimental protocol requires adherence to local institutional guidelines for laboratory safety and ethics.

## Graphical abstract

**Figure:**
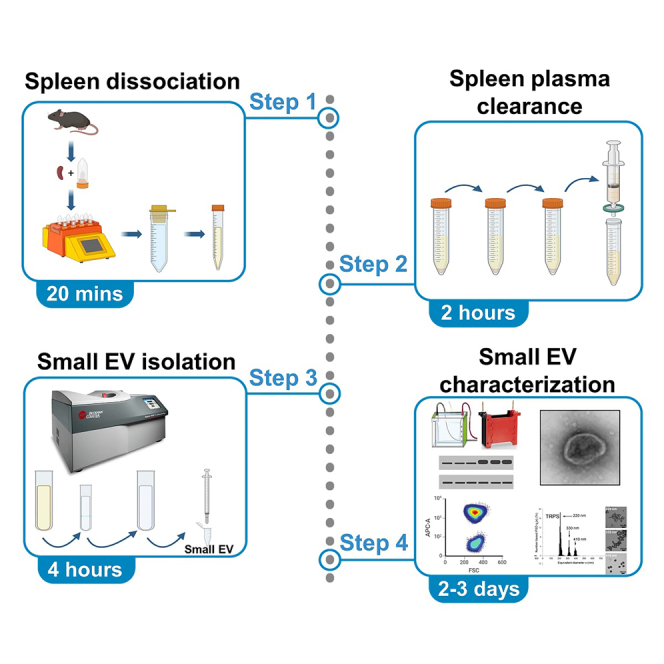

## Before you begin

The protocol described here has been optimized to isolate small extracellular vesicles (sEV) from the spleen of the pre-clinical murine transgenic models of leukemia Eμ-TCL1. The protocol can also be applied to spleens derived from control mice (C57Bl/6) injected with TCL1 cells (adoptive transfer models; AT) and healthy control mice (HC).

1. Identify the animal that has reached an adequate stage of the disease (typically a leukemic Eμ-TCL1 mouse above 50 weeks of age).
2. Prepare sterile surgical instruments and surface to dissect the mouse and harvest the spleen.
3. Prefill a gentleMACS tube (1 per spleen) with 5 mL (max 10 mL if the spleen is of considerable size) of cold phosphate-buffered saline (PBS) without Ca^2+^ and Mg^2+^ and keep it on ice.
4. Prepare the 17% OptiPrep needed for sEV isolation using ultracentrifugation.

***Note:*** The preparation of all buffers and reagents must be completed prior to euthanizing the mouse to ensure a swift harvesting process. All solutions should be filtered with a 0.22 μm filter before use. Spleens should be maintained at low temperature following the harvest.

**CRITICAL:** To ensure that sufficient amounts of sEV are collected for subsequent analyses, it is recommended that leukemic mice (transgenic or AT) reach at least 70% of CD5^+^CD19^+^ leukemic cells in peripheral blood (PB), measurable by flow cytometry.^2^

### Institutional permissions

All experiments involving laboratory animals were conducted in a pathogen-free animal facility with the approval of the Luxembourg Ministry for Agriculture. Mice were treated in accordance with the European Union guidelines.

It is essential to obtain permission to perform animal experimentation in due time since application revision, editing and granting might require several weeks prior to initiating the experiment.

### Spleen dissociation and spleen plasma clearance

**Timing: 10 min spleen dissociation; 2 h spleen plasma clearance**

5. Mechanical dissociation and filtration.
6. Clearance of spleen plasma through progressively higher centrifugation speeds.

***Note:*** Upon spleen plasma clearance, it is possible to immediately proceed to the next step (sEV isolation) or freeze the sample at −80°C.

### Small EV isolation

**Timing: 4 h sEV isolation and purification**

7. Collect sEV and soluble proteins using ultracentrifugation.
8. Separate sEV from soluble proteins and other contaminants using ultracentrifugation coupled with sucrose gradient.
9. Collect purified sEV using ultracentrifugation.

**CRITICAL:** sEV obtained in points 3 and 5 need to be completely resuspended in filtered PBS before proceeding to the next steps.

## Key resources table

**Table:** 

REAGENT or RESOURCE	SOURCE	IDENTIFIER
**Biological samples**		

*Eμ-TCL1* mouse (above 50 weeks of age) spleen		

**Chemicals, peptides, and recombinant proteins**		

OptiPrep	STEMCELL Technologies	07820
BioTracker MemBright 488 live cell dye	Merck	SCT083
Murine anti-CD63 PE antibody, clone REA563	Miltenyi Biotec	130-108-893
Murine anti-CD81 APC antibody, clone EAT2	Miltenyi Biotec	130-102-630
Dulbecco’s phosphate-buffered saline [-] CaCl_2_ [-] MgCl_2_	Thermo Fisher Scientific	14190-094

**Experimental models: Organisms/strains**		

Mouse: *Eμ-TCL1* (on C57BL/6 background)	Pr. Carlo Croce (OSU, OH, USA)	MGI:3527221

**Other**		

gentleMACS dissociator	Miltenyi Biotec	130-093-235
Ultracentrifuge Optima MAX-XP	Beckman Coulter	393315
MLA-55 fixed-angle rotor	Beckman Coulter	393203
MLS-50 swinging-bucket rotor	Beckman Coulter	367280
0.22 μL syringe filter	Starlab	E4780-1223
0.45 μL filter (4 mm) polyvinylidene difluoride (PVDF)	EMD Millipore	SLHVR04NL
Cell strainer 40 μm nylon	Corning	352340
gentleMACS C tubes	Miltenyi Biotec	130-093-237

## Materials and equipment

### Chemical solutions

10% Sodium dodecyl sulfate (SDS) (10 g SDS, to 100 mL with ddH_2_O).

5% Bovine Serum Albumin (BSA) (1 g BSA, to 20 mL with TBS-T 1X).

5% milk (1 g milk powder, to 20 mL with TBS-T 1X).

### Buffers

**Table:** 17% OptiPrep solution

Solution A (pH 7.4)		

Reagent	Final concentration	

Sucrose	0.25 M	
Ethylenediaminetetraacetic acid (EDTA)	6 mM	
Tris hydrochloride (Tris HCL)	60 mM	

**Working Solution (WS1)**		

**Reagent**		**Amount**

60% OptiPrep	50%	5 volumes
Solution A (pH 7.4)	0.167 X	1 volume

**Homogenization medium (HM1; pH 7.4)**		

**Reagent**	**Final concentration**	**Amount**

Sucrose	0.25 M	
Ethylenediaminetetraacetic acid (EDTA)	1 mM	
Tris hydrochloride (Tris HCL)	10 mM	

Store at 4°C for up to 3 months.

***Note:*** To prepare the final solution, dilute Working Solution 1 (WS1) with Homogenization medium HM1 to obtain a final concentration of 17%. The final diluted solution can be stored at 4°C for up to 3 months.

**Table:** Sample buffer 4X

Reagents	Final concentration	Amount
Tris HCl 1 M (pH-6.8)	0.1 M	5 mL
Sodium Dodecyl Sulfate (SDS) 10%	4%	20 mL
Glycerol 87%	35%	20 mL
Bromophenol Blue 1%	0.01%	500 uL
B-mercaptoethanol	10%	5 mL

Store at −20°C for up to 1 year.

**Table:** Tris-buffered saline-Tween (TBST) 1X (pH 7.4)

Reagents	Final concentration	Amount
NaCl	0.15 M	8.76 g
Tris	50 mM	6.05 g
TWEEN 20	0.1%	1 mL
ddH_2_O		q.s. 1 L

Store at 4°C for up to 1 month.

**Table:** Transfer buffer 1X

Reagents	Final concentration	Amount
Tris	50 mM	6.05 g
Glycine	200 mM	28.84 g
Ethanol or methanol	20%	200 mL
ddH_2_O		q.s. 1 L

Store at 4°C for up to 1 month.

**Table:** Running buffer 1X

Reagents	Final concentration	Amount
Tris	50 mM	6.05 g
Glycine	200 mM	28.84 g
SDS 10%	0.1%	10 mL
ddH_2_O		q.s. 1 L

Prepare immediately before the experiment.

## Step-by-step method details

### Spleen dissociation and spleen plasma clearance

**Timing: 10 min spleen dissociation; 2 h spleen plasma clearance**

**Figure 1:**
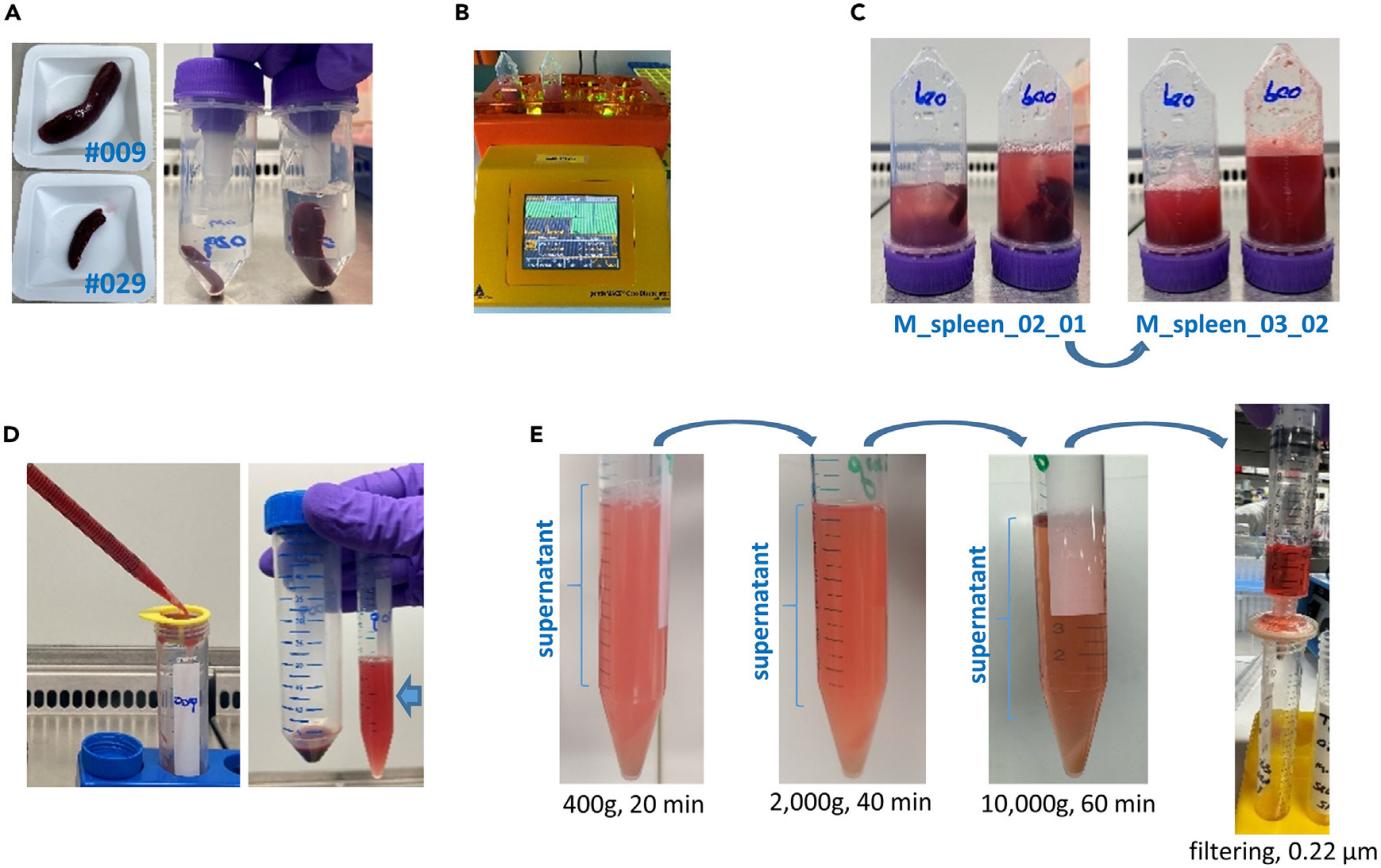
Spleen dissociation and spleen plasma clearance (A) Examples of two leukemic spleens (#009 and #029) outside and inside gentleMACS C tubes with different level of PBS based on spleen size. (B) Spleen disruption program running at the gentleMACS machine. (C) GentleMACS C tubes with leukemic spleens after program M_spleen_02_01 (left photo) and subsequent program M_spleen_03_02 (right photo). (D) Filtering of the dissociated spleen in a 100 μm cell strainer (left photo) and pellet plus supernatant, marked with an arrow, after the first centrifugation at 400 g (right photo). (E) Example of the 15 mL conical tubes after each of the serial centrifugations, and setting of the filtering of the cleared supernatant.

This step allows to mechanically dissociate the murine spleen and to progressively remove unwanted components (cells, large microvesicles and apoptotic bodies) from the supernatant (Figures 1A–1D).

1. At the gentleMACS machine:
  a. Maintain the freshly isolated spleen in cold PBS on ice.
  b. Place the spleens in cold PBS (5–10 mL) inside the gentleMACS C tubes, ensure that the final volume remains between 5 to 10 mL, and insert the tubes in the gentleMACS machine.
  c. Use the pre-existing program *M_spleen_02_01* for a superficial rupture of the spleen.
  d. Immediately follow with the pre-existing program *M_spleen_03_02* to fully dissociate and homogenize the tissue.
  e. Utilize a 100 μm cell strainer to strain the dissociated spleen plasma in a 15 or 50 mL conical tube, removing any tissue clumps and large debris.

***Note:*** Due to the viscosity of the homogenized tissue, the cell strainer could easily clog when using low volumes and 15 mL tubes, making the process of filtration more difficult. If starting with 5 mL volume, up to 10 mL of cold PBS without Ca^2+^ and Mg^2+^ can be added to improve the filtration process. Furthermore, the flat side of a syringe plunger can be used to gently move the homogenized tissue on the filter, mechanically supporting the filtration process.

2. Spin the 50 or 15 mL conical tube containing the spleen plasma in a centrifuge pre-cooled to 4°C (Figure 1E).
  a. Spin the tube at 400 × *g*, 5 min, 4°C. Immediately transfer the supernatant to a new 15 mL conical tube. The cells (splenocytes) can be repurposed.
  b. Spin the tube at 400 × *g*, 20 min, 4°C. Immediately transfer the supernatant to a new 15 mL Eppendorf tube. This process allows to further remove remaining cells.
  c. Spin the tube at 2,000 × *g*, 40 min, 4°C. Immediately transfer the supernatant to a new 15 mL Eppendorf tube. This process allows to remove large vesicles (e.g., microvesicles).
  d. Spin the tube at 10,000 × *g*, 60 min, 4°C. Immediately transfer the supernatant to a 10 mL syringe. This process allows to remove small contaminant particles (e.g., apoptotic bodies).
  e. Filter the cleared spleen plasma through a 0.22 μm filter (13 mm) in a new 15 mL Eppendorf tube.
  f. Immediately proceed to the next major step (sEV isolation).

**Pause point:** Alternatively, the cleared spleen plasma can be stored at −80°C for several months.

***Note:*** To avoid any potential contamination of the supernatant by the pelleted material, always transfer the supernatant to a new 15 mL conical tube immediately after each centrifugation step, leaving a small volume (∼ 200 μL) above the pellet, to leave the pellet undisturbed.

**CRITICAL:** Upon harvesting, the spleen should be immediately collected in a gentleMACS C tube prefilled with 5–10 mL (depending on spleen size) of cold PBS without Ca^2+^ and Mg^2+^ and kept on ice during the entire procedure.

### Small EV isolation

**Timing: 4 h sEV isolation and purification**

**Figure 2:**
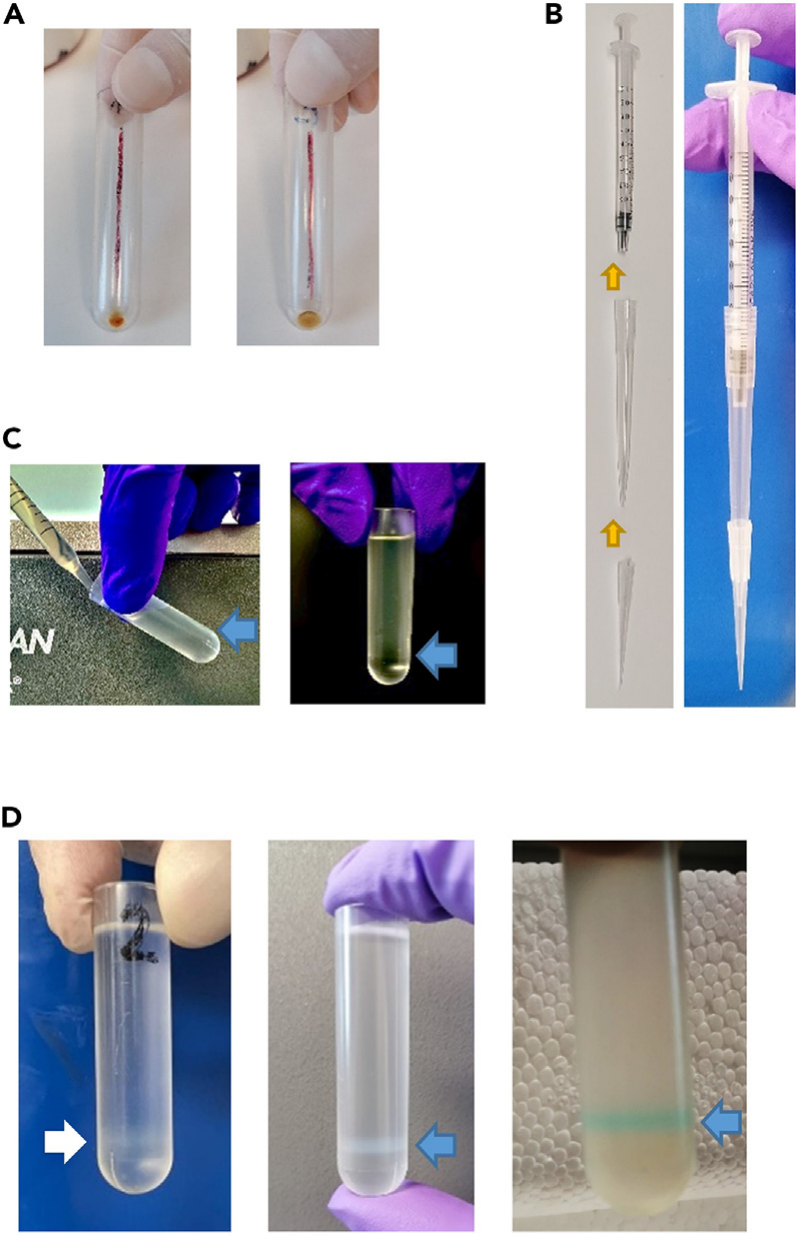
Small EV isolation from the cleared spleen plasma (A) Representative pellet of sEV after the first ultracentrifugation at 110,000 g for 70 min. (B) Building of the 1 mL syringe in combination with a p200 tip and a p1000 tip to resuspend the sEV pellet. (C) Laying process on the 17% OptiPrep (cushion). Arrows indicate the interphase formed. (D) Pale ring, showing the interphase after the cushion ultracentrifugation, containing the sEV (indicated with an arrow). Different intensities of the pale ring may be observed in different preparations. In the last picture, sEV were pre-stained with a membrane dye.

This step facilitates the collection of sEV through ultracentrifugation. Simultaneously, a sucrose gradient-based cushion ensures elevated vesicle purity by eliminating soluble contaminants. Ultrafiltration steps further increase purity and homogeneity of the preparation (Figure 2).

3. Transfer the cleared spleen plasma (min 7 mL/max 8.5 mL) to a 9 mL thick wall ultracentrifuge tube (16 × 76 mm).

***Note:*** Draw a vertical line on one side of the tube and place this line towards the outside of the rotor. Upon ultracentrifugation, the sEV pellet will localize under this line.

4. Centrifuge at 110,000 × *g*, 70 min, 4°C (acceleration set at 0 and brake set at 9) using a MLA-55 (ultra-fixed angle) rotor and an ultracentrifuge Optima MAX-XP.

**CRITICAL:** When the centrifuge stops, proceed immediately to the next step to avoid sEV pellet dispersion.

5. Discard the supernatant by decanting into a liquid waste disposal. To accomplish this, invert the tube once, position it upside-down, and carefully place it on a wipe paper until all the liquid is absorbed.

**CRITICAL:** Ensure that the drawn line, where the pellet is located, is directed upward to avoid resuspension of the sEV pellet and losing material (Figure 2A).

6. Add 1 mL cold PBS without Ca^2+^ and Mg^2+^ to the sEV pellet.

***Optional:*** If the labelling of sEV with a fluorescent probes is needed, use 1 mL cold PBS without Ca^2+^ and Mg^2+^ supplemented with the dye (e.g. BioTracker MemBright 488 Live Cell Dye, later called MB) for a final concentration of 200 nmol/L.

7. The pellet needs to be mechanically resuspended. Pipette up and down using a 1 mL syringe in combination with a 200 μL pipette tip and a 1 mL pipette tip (Figure 2B).

***Note:*** Leaving the sEV pellet incubating in cold PBS without Ca^2+^ and Mg^2+^ for about 30–40 min at 4°C can help the mechanical dispersion.

***Optional:*** If using MB to fluorescently label sEV, leave the resuspended sEV on ice for at least 15 min before proceeding with the next step.

**CRITICAL:** In this step, it is crucial to avoid direct contact between the sEV pellet and the pipette tip, as part of the pellet may adhere to the tip, making the resuspension procedure more difficult. Additionally, it is essential to minimize the formation of bubbles, as they can further add to the difficulty and potentially result in the loss of material.

**CRITICAL:** Before proceeding to the next step, ensure that the pellet is completely dispersed in solution and there should not be any visible fragments.

8. Add an addition of 3 mL cold PBS without Ca^2+^ and Mg^2+^ to the tube.
9. Prefill a 5 mL thin wall polyallomer ultracentrifuge tube (13 × 51 mm) with 1 mL 17% OptiPrep (cushion).

***Note:*** Prepare the 17% OptiPrep in advance (can be stored at 4°C for up to 6 months).

10. Gently layer the resuspended sEV (4 mL) on top of the cushion. Troubleshooting 1.

**CRITICAL:** It is important that the layering process is slow and consistent to avoid mixing the two phases. This can be performed with any pipetting tool allowing low speed liquid dispensing such a pipette boy with release speed set at 0 or a 1000 μL manual pipette with a plunger system requiring lower force (Figure 2C).

11. Centrifuge at 100,000 × *g*, 70 min, 4°C (acceleration set at 0 and brake set at 9) using a MLS-50 (swinging-bucket) rotor and an ultracentrifuge Optima MAX-XP.

**CRITICAL:** When the centrifuge stops, proceed immediately to the next step to avoid sEV pellet dispersion

***Note:*** A pale ring (interphase) containing the sEV should be visible below half of the total volume in the tube (Figure 2D). Note that the ring will be slightly colored in the case of fluorescence-labelled EVs.

12. Gently remove 3 mL of solution above the interphase using a pipette.

**CRITICAL:** Aspirate from the surface and move below the solution level, avoiding disturbing the ring and proceed to aspirate the sEV from the interphase.

13. Collect the interphase containing the sEV (max 1 mL) using a pipette.

**CRITICAL:** To collect 1 mL of sEV from the interphase, aspirate from the pale ring and move below following the solution level and being careful to not reach the bottom of the tube. Indeed, a pellet containing membrane debris and contaminant material can sometimes be observed at the bottom of the tube.

14. Transfer the sEV (1 mL) to thick wall ultracentrifuge tube (16 × 76 mm) and add 7.5 mL of cold PBS without Ca^2+^ and Mg^2+^.

***Note:*** Place the line drawn on the tube forwards the outside of the rotor.

15. Centrifuge at 110,000 × *g*, 70 min, 4°C (acceleration set at 0 and brake set at 9) using a MLA-55 (ultra-fixed angle) rotor and an ultracentrifuge Optima MAX-XP.

**CRITICAL:** When the centrifuge stops, proceed immediately to the next step to avoid sEV pellet dispersion

16. Eliminate the supernatant by decanting the supernatant in a liquid waste disposal.

**CRITICAL:** Ensure that the drawn line, where the pellet is located, is directed upward to avoid loss of material via resuspension of the sEV pellet.

17. Resuspend the sEV pellet in 500 μL (max 1 mL) cold PBS without Ca^2+^ and Mg^2+^.
18. The pellet should be dispersed using a 1 mL syringe in combination with a yellow (p200) and blue (p1000) tips (as schematized in Figure 2B). Continue pipetting until the pellet has been completely resuspended.

**CRITICAL:** Do not make direct contact with the pellet when using the pipette tip, and avoid generating bubbles. Before moving to the next step, the pellet should be completely resuspended with no visible aggregates in the solution.

19. Filter the sEV through a 0.45 μL filter (4 mm) using a 1 mL syringe.

***Note:*** If the isolated sEV are immediately used for *in vitro* or *in vivo* experiments, filter the preparation once more through a 0.22 μm filter (4 mm) to further reduce the risk of contaminations. Troubleshooting 2.

**CRITICAL:** Depending on the concentration of sEV, the filter can easily clog. Do not force the filtration or the added pressure will result in the detachment of the filter from the syringe, causing significant loss of material. Rather, change the filter as soon as the flow through rate reduces, requiring additional pressure.

***Note:*** While changing the filter, the remaining sEV volume on the inside of the previous filters can be collected with a pipette.

20. Save a small aliquot of filtered sEV for protein measurement.

***Note:*** The exact volume depends on the assay you will use. For instance, save 4.5 μL if using the Bradford protein quantification assay.^3^

21. Aliquots (100–200 μL) of the filtered sEV in Eppendorf tubes can be stored at −80°C.

***Note:*** Freezing and thawing process disrupt sEV structure. Having multiple aliquots allow several assays and experiments to be performed without loss of sEV material and properties.

## Expected outcomes

After performing our isolation and purification protocol, researchers can expect to obtain a purified sEV population derived from both leukemic cells and microenvironment, called LME-sEV. The effectiveness and efficacy of this protocol have been validated extensively in murine spleen samples. From our experience, the amount of LME-sEV from 1 g of leukemic spleen can range from 550 to 2300 μg.

**Figure 3:**
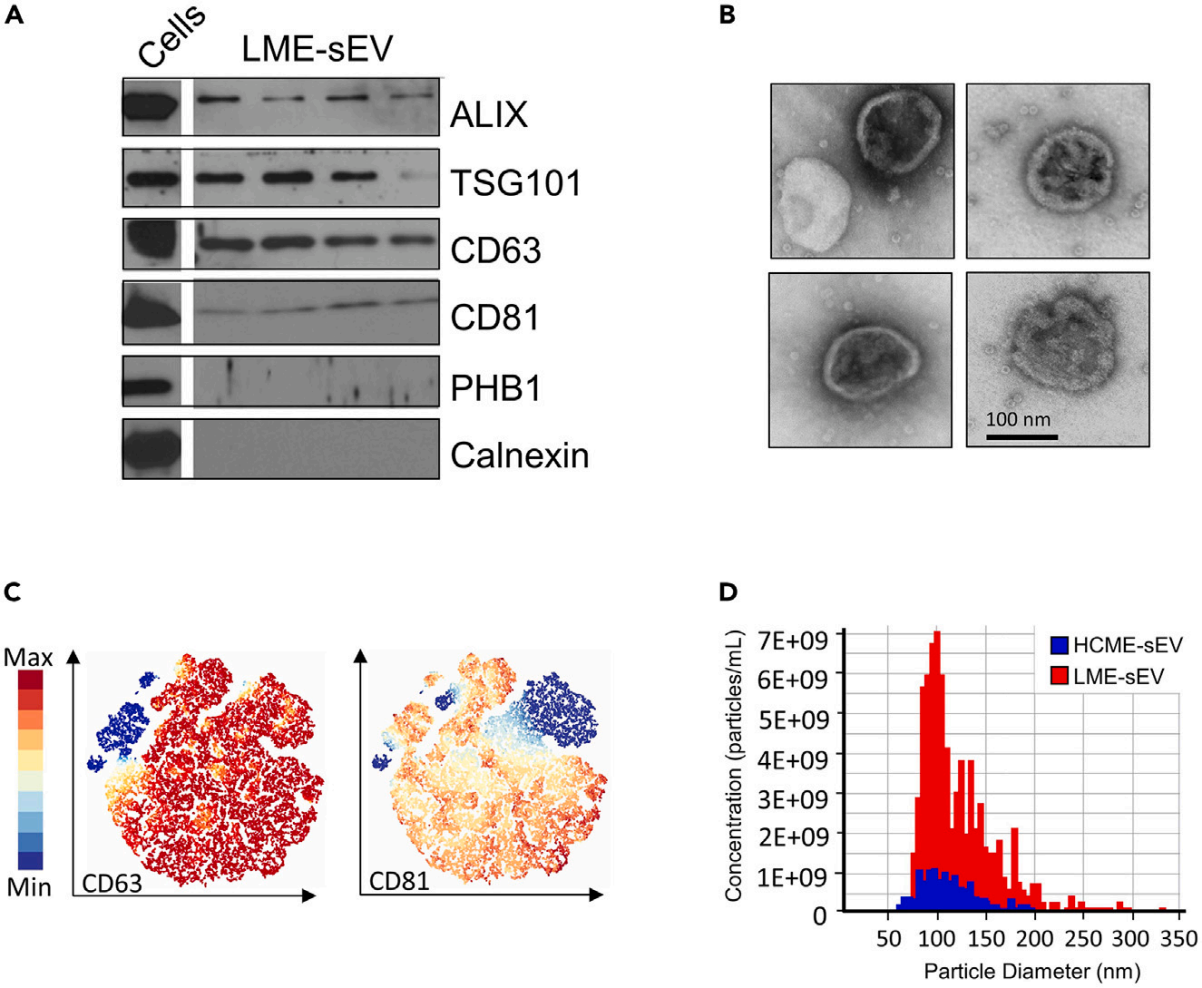
Small EV characterization (A) Western blot (WB) depicting the enrichment of EV-specific biomarkers such as Alix, TSG-101, CD63 and CD81, from 4 different leukemic sEV preparations and whole cells. (B) Representative electron microscopy images of purified leukemic sEV. (C) HSNE clustering analysis of MB488+ LME-sEVs based on positivity for CD63 and CD81, measured by bead-free Flow Cytometry. (D) Tunable resisting pulse sensing (TRPS) results showing the particle-size distribution profiling of purified leukemic sEV, obtained from the Exoid machine (Izon Science).

To quantify the amount of sEV, and to evaluate their purity, we recommend multiple strategies as previously indicated by MISEV 2023.^4^ Western blot (WB) can be used to verify the enrichment of EV-specific biomarkers such as Alix, TSG-101, CD63 and CD81 involved in sEV biogenesis. Including negative controls, such as prohibitin 1 (Phb1) and Calnexin, is also essential to confirm the absence of contaminants (Figure 3A)^1^^,^^3^ Detection of non-EV-specific markers may suggest potential contamination and would require further attention during the protocol.

To assess the overall sEV morphology and integrity, electron microscopy (EM) imaging can be utilized. This allows researchers to confirm the presence of sEV within the sample and obtain single particle measurements, such as diameter (Figure 3B).

Bead-free flow cytometry analysis can be performed on the purified sEV preparations. Acquisition can be done with a NovoCyte Quanteon Flow Cytometer (Agilent) equipped with a 0.22-μm filter for the sheath fluid to reduce electronic noise, setting the instrument to a minimum flow rate (5 μL/min). All solutions should be filtered with a 0.22 μm filter before acquisition. In Figure 3C we show a representative hierarchical stochastic neighbor embedding (HSNE) clustering analysis, performed with Cytosplore software,^5^ for the presence of CD63 and CD81 markers in the purified sEV preparations stained with the MB488 dye.

For further in-depth particle-size distribution profiling, tunable resisting pulse sensing (TRPS) can be performed with the Exoid instrument (Izon Science) (Figure 3D) or any other device allowing size and ζ-potential measurement.

This protocol has been optimized for murine spleen samples, showing excellent results in isolating highly pure sEV. We anticipate that this protocol can be applied to other tissue samples and various experimental settings, yielding similar results and outcomes. Nevertheless, researchers may need to adapt the protocol for different tissue types and are encouraged to refer to updated guidelines from organizations such as the International Society for Extracellular Vesicles (ISEV) for additional information on characterizing different classes of EV.

Overall, this protocol provides a reliable and effective method to isolate and characterize sEV, which can be valuable for various downstream applications, including studying intercellular communication and potential diagnostic and therapeutic applications.^6^

## Limitations

The original scope of our protocol was to capture the complexity and heterogeneity of sEV in the leukemia microenvironment. Nevertheless, from the biological point of view, when testing the effect of these preparations, one possible limitation is the inability to accurately identify the cell of origin that released these sEV. Although the vast majority of the sEVs in the pool originate from B leukemic cells, we cannot predict the incidence of other cell types in the total composition of these heterogeneous preparations. This is not the case when purifying sEV derived of specific cell lines *in vitro*.^7^

**SUGGESTION:** If the exact composition is important to understand a biological phenomenon or to answer a specific question, we recommend to perform a single sEV immunophenotyping to measure the proportion of the sEV preparation carrying specific markers related to the different cells of origin present in the organ analyzed. This method is possible with a small range of flow cytometers, including the NovoCyte Quanteon flow cytometer (Agilent) or the ImageStreamX Mk II Imaging Flow Cytometer (Cytek Amnis).

Our UC protocol, when used without cushion gradient (during the first and last round of UC), has the potential to deform or fuse sEV. The use of fused sEV is highly reduced by the ultrafiltration and cushion gradient steps. However, UC isolation can still potentially damage the integrity and yield of exosomes, therefore leading to reduced sEV functionality.^8^^,^^9^^,^^10^

For our intended purposes, LME-sEV still exhibited considerable activity, nevertheless, it is plausible that some functionalities may have been reduced or even lost. Given this known limitation, it might be valuable to explore our protocol in combination with alternative isolation strategies. Indeed, UC can be used with large quantities of starting material and when partial loss is considered acceptable.

**SUGGESTION:** Alternative methods for isolating and purifying sEV have emerged in recent years. Techniques such as size-exclusion chromatography (SEC), ultrafiltration, and antibody-based capture may be preferred when handling small volumes or when the use of untouched vesicles is important.^4^^,^^11^

## Troubleshooting

### Problem 1

When laying the resuspended sEV (4 mL) on the top of the cushion (1 mL 17% OptiPrep), the 2 phases mix making them indistinguishable.

### Potential solution

• This step is essential to obtain highly purified sEV. Thus we recommend transferring back the 5 mL of PBS containing sEV to a 9 mL thick wall ultracentrifuge tube, fill up with PBS, and repeat step 4 to isolate sEV again.
• Furthermore, we recommend identifying the reason of the unsuccessful procedure and, if needed, train on slowly laying 4 mL PBS on top of 1 mL 17% OptiPrep.
• The OptiPrep solution does not have the correct density. Repeat the preparation under materials and equipment section.

### Problem 2

During the final step of sEV filtering, the filter is getting clogged due to the high pressure applied, risking collapse and subsequent sample loss.

### Potential solution

• If the preparation does not go smoothly after pressuring (firmly but not forcing the system), step 18 should be repeated and done more thoroughly. Incubations on ice for 10 min, vortexing and continuing repetitions of step 18 are also encouraged.
• Alternatively, we recommend diluting the sample more, adding 250–500 μL of PBS, and afterward repeat these steps.

## Resource availability

### Lead contact

Further information and requests for resources and reagents should be directed to the lead contact, Etienne Moussay (etienne.moussay@lih.lu).

### Technical contact

Further technical requests should be directed to the technical contact, Ernesto Gargiulo (ernesto.gargiulo@gmail.com).

### Materials availability

This study did not generate new unique materials or reagents.

### Data and code availability

This study did not generate new databases or code.
